# Psychosocial Correlates of Network Orientation: A Cross-National Comparison of University Students in Mexico and Russia

**DOI:** 10.3390/bs16081299

**Published:** 2026-07-30

**Authors:** Irina Melnikova-Abramova, Iván Delgado-Enciso, Gustavo A. Hernández-Fuentes, Svetlana E. Strogova, Elena N. Arbuzova, Osiris G. Delgado-Enciso, Jessica C. Romero-Michel, Verónica M. Guzmán-Sandoval, Mario Ramírez-Flores, Fabian Rojas-Larios, Carmen A. Sanchez-Ramirez, Alejandrina Rodríguez-Hernandez, Valery Melnikov

**Affiliations:** 1 Psychology Faculty, Saint Petersburg State University, Makarova Embankment, 6, St. Petersburg 199034, Russia; virusdrawss@gmail.com; 2 Department of Molecular Medicine, School of Medicine, University of Colima, Colima 28040, Mexico; ivan_delgado_enciso@ucol.mx (I.D.-E.); ghfuentes@ucol.mx (G.A.H.-F.); 1933osiris@gmail.com (O.G.D.-E.); mario_ramirez@ucol.mx (M.R.-F.); frojas@ucol.mx (F.R.-L.); carmen_sanchez@ucol.mx (C.A.S.-R.); arodrig@ucol.mx (A.R.-H.); 3 State Cancerology Institute of Colima, Health Services of the Mexican Social Security Institute for Welfare (IMSS-BIENESTAR), Colima 28085, Mexico; 4 Robert Stempel College of Public Health and Social Work, Florida International University, Miami, FL 33199, USA; 5 Faculty of Chemical Sciences, University of Colima, Coquimatlan 28400, Mexico; 6 Department of Adjustment Disorders and Trauma, Institute of Child Psychiatry of the Federal State Budgetary Scientific Institution “Russian Mental Health Research Centre”, Kashirskoe Shosse 34, Moscow 115522, Russia; 2607@1090393.ru; 7 Department of Crisis and Extreme Situation Psychology, Faculty of Psychology, Saint Petersburg State University, Makarova Embankment 6, St. Petersburg 199034, Russia; e.arbuzova@spbu.ru; 8 Faculty of Law, University of Colima, Colima 28040, Mexico; jessica_romero@ucol.mx; 9 School of Psychology, University of Colima, Colima 28040, Mexico; gus_vero@ucol.mx

**Keywords:** help-seeking, network orientation, generalized trust, self-esteem, interpersonal sympathy, Mexico, Russia

## Abstract

Network orientation reflects beliefs about whether seeking help from others is desirable, safe, and useful. Cross-national studies have often focused on differences in the mean levels of negative network orientation; however, similar help-seeking scores may conceal distinct underlying psychosocial structures. Methods: A cross-sectional study was conducted among 676 university students from Mexico (*n* = 422) and Russia (*n* = 254). Participants completed the Network Orientation Scale (NOS), Rosenberg Self-Esteem Scale, Generalized Trust Scale, and Interpersonal Sympathy Scale. Mean comparisons, Pearson correlations with Fisher’s r-to-z tests, and multivariable linear interaction models were performed. Results: Mean NOS levels were similar across countries, but the psychosocial correlates differed substantially. Russian students reported higher generalized trust and higher self-esteem, whereas interpersonal sympathy was comparable between the countries. Greater interpersonal sympathy was associated with higher network avoidance in both populations, while generalized trust was associated with lower network avoidance, with a stronger effect in Russia. Self-esteem was not significantly associated with network orientation in Mexico but was significantly associated with lower network orientation in Russia. Continuous multivariable interaction models demonstrated a significant self-esteem × country interaction. Men showed higher network orientation than women in both countries. Conclusions: Although Mexican and Russian university students showed similar average levels of negative network orientation, the psychosocial structure underlying help-seeking differed across contexts. These findings suggest that help-seeking tendencies should not be interpreted solely through mean comparisons, but also through the psychosocial configurations shaping support-seeking within each cultural setting.

## 1. Introduction

Seeking support from others is a fundamental component of human social functioning and psychological adaptation. However, individuals differ substantially in their willingness to mobilize social support when facing distress or adversity. Network orientation reflects predisposition or attitudinal stance whether seeking help from others is desirable, safe, and useful and has been recognized as an important determinant of help-seeking behavior (Cohen & Wills, 1985).

The capacity to seek and provide help constitutes a foundational prosocial mechanism that has shaped human survival and resilience throughout evolution (Penner et al., 2005; Twenge et al., 2019).

Given the diversity of factors involved, individuals differ in their willingness to mobilize social support when facing distress or adversity. Self-esteem, generalized trust, and interpersonal sympathy represent relevant psychosocial dimensions associated with this construct, although their effects may not operate uniformly across sociocultural contexts. According to Luhmann et al. (2017), trust functions as a mechanism for reducing social complexity, allowing individuals to act under conditions of uncertainty. In this sense, generalized trust may decrease the perceived interpersonal risks associated with seeking help, whereas its absence may hinder social interaction and support mobilization.

Self-esteem has also been identified as an important psychological factor associated with help-seeking behaviors (Bandura, 2000; Porter & Schumann, 2018). Self-esteem, understood as a global affective evaluation of the self (Rosenberg, 1965), may interfere with help-seeking at both extremes of its spectrum. Low self-esteem can discourage individuals from asking for support due to fears of being perceived as incompetent. High self-esteem, depending on the context, may be associated with self-efficacy and a preference for independent problem-solving or with social confidence, interpersonal effectiveness, and a reduced perceived risk of stigma when expressing a need (Vogel & Wester, 2003). Maladaptive high self-esteem (e.g., narcissistic traits) may foster a negative orientation toward support networks by limiting both the expression and reception of help-related communication. In both extreme cases, relational exchange processes become more difficult for the individual and their social network, thereby contributing to social fragmentation (Ostrom, 1990). Furthermore, the influence of self-esteem on help-seeking decisions may itself be shaped by cultural context. Similarly, interpersonal sympathy may encourage closeness and prosocial engagement but paradoxically may also inhibit support-seeking due to concerns about burdening others.

Alongside individual psychological factors, cultural belonging also shapes attitudes toward mutual support. Among cultural dimensions, the degree of individualism has been identified as a key determinant of mutual aid behaviors (Hofstede et al., 2010; Jeng, 2024). Likewise, communicative context, as conceptualized by Hall (2003), influences patterns of interaction that shape how social support is mobilized. Consequently, cross-cultural comparative studies may help clarify the extent to which cultural context continues to influence support-seeking in an increasingly globalized world.

According to Schwartz (1992), cultural specificity does not derive from possessing unique values but rather from the distinctive prioritization societies assign to shared values in order to address collective challenges. From this perspective, culture becomes a measurable value system that shapes how individuals perceive trust, support, and their place within the community. This integrative framework allows intercultural differences to be interpreted not as arbitrary divergences, but as distinct responses to universally shared human challenges.

The present study applies psychometric instruments designed to evaluate characteristics associated with or conditioning the disposition toward mutual aid. The same population group was examined in two societies that differ substantially in contextual communication patterns (Hall, 2003), collectivism, and uncertainty avoidance (Hofstede et al., 2010). From an operational psychological perspective, willingness to seek support can be related to network orientation, a construct encompassing beliefs and expectations regarding whether turning to others is useful, appropriate, and safe (Vaux et al., 1986). High scores on the Network Orientation Scale (NOS) indicate greater network avoidance (i.e., a more negative orientation toward seeking social support).

Accordingly, this study proposed a comparative analysis of self-esteem, interpersonal sympathy, generalized trust, and predisposition to seek help among university students from Mexico and Russia. These groups were selected because they represent distinct sociocultural contexts that have been described as differing in communication patterns and broader historical, institutional, and social characteristics, allowing us to examine whether locally embedded cultural norms continue to influence help-seeking attitudes, even among millennial university students—a demographic often characterized as sharing a globalized identity (Rouse et al., 2022). However, these contextual characteristics were not directly measured in the present study and are considered only as conceptual background for the cross-national comparison. The main objective was to determine whether the associations between psychosocial factors and network orientation differed between countries, rather than to attribute these differences to any specific cultural explanation. Accordingly, the present study was designed to compare observed associations rather than to identify causal or cultural mechanisms underlying help-seeking behavior.

## 2. Materials and Methods

### 2.1. Study Design and Sample

This study consisted of a cross-sectional comparative analysis using a cleaned final database restricted to participants from Mexico and Russia. The analyzed sample included 676 young adults aged 18–29 years, of whom 422 were from Mexico and 254 from Russia. A non-probabilistic convenience sampling strategy was employed. Participants were recruited through announcements distributed via commonly used social media platforms (e.g., Facebook, WhatsApp, Telegram, and VK in Russia, as appropriate), together with institutional e-mail and other communication channels by collaborators from the participating universities in each country. Recruitment followed the same general protocol in both settings, whereby university students were invited to complete an anonymous online questionnaire voluntarily after providing electronic informed consent.

The present study formed part of an ongoing research program examining sociocultural and mental health factors among university students. The overall research program was approved by the Research Ethics Committee of the State Cancer Institute (approval number CEICANCL230317-ASEXCCC-06; 2 March 2017). The protocol remained active throughout the study period, and the data analyzed in the present cross-sectional investigation were collected between 2023 and 2024 under this approved research protocol. Inclusion criteria consisted of being between 18 and 29 years of age, being born in Mexico and currently enrolled in an undergraduate program at a Mexican university, or being born in Russia and currently enrolled in an undergraduate program at a Russian university.

Mexican participants were recruited from the University of Colima, Universidad Michoacana de San Nicolás de Hidalgo, Tecnológico de Colima, Benemérita Universidad Autónoma de Puebla, and the University of Guadalajara. Russian participants were recruited from Saint Petersburg State University, Volgograd State Socio-Pedagogical University, Siberian Federal University, MIREA—Russian Technological University, Perm State Humanitarian Pedagogical University, Ural Federal University, and Saratov State University. Because recruitment relied on voluntary participation through collaborating universities, detailed information regarding participants’ academic disciplines and socioeconomic characteristics was not collected uniformly across institutions and was therefore not available for analysis. Consequently, the findings should be interpreted as reflecting associations observed within two convenience samples of university students rather than nationally representative populations. Participants with incomplete surveys were excluded from the analyses. Furthermore, although the participating universities served primarily as recruitment sites and the objective of the study was to compare psychosocial associations between countries, clustering by institution was not explicitly modeled. This should be considered when interpreting the findings, as some within-university correlation may have remained unaccounted for. The study was based on non-probabilistic convenience samples of university students and therefore should not be interpreted as representative of the broader populations of either country.

### 2.2. Measures

Network orientation was assessed using the 20-item Network Orientation Scale (NOS), developed by Vaux et al. (1986). Higher NOS scores indicate greater resistance to seeking or accepting interpersonal support. Self-esteem was measured using the 10-item Rosenberg Self-Esteem Scale (RSES) (Gnambs et al., 2018; Robins et al., 2001). The RSES was administered using identical four-point response categories in both language versions, and identical scoring rules were applied across countries. Complete item wording, response options, and scoring procedures for all instruments are provided in the Supplementary Materials. Generalized trust was evaluated using the 6-item Generalized Trust Scale developed by Yamagishi and Yamagishi (1994). Interpersonal sympathy was assessed using the 15-item Linking People Scale (LPS), originally developed by Filsinger (1981), which was used as a measure of interpersonal sympathy.

Participants from Mexico completed the Spanish-language versions of the questionnaires, whereas participants from Russia completed the corresponding Russian-language versions administered by the collaborating research team at Saint Petersburg State University (for the NOS and Linking People Scale) and taken from validated published versions (for the Rosenberg and Yamagishi scales). The original sources and, where available, the corresponding language adaptation studies are cited for each instrument (Arbuzova & Gorbatov, 2025; Martín-Albo et al., 2007; Zolotareva, 2020; Vlasenko, 2022; Montoro et al., 2014)) NOS and Linking People Scale were translated into Spanish and linguistically and statistically validated by our group. Complete item wording, response options, and scoring procedures for all instruments in English, Spanish, and Russian are provided in the Supplementary Materials. For the psychometric analyses, item-level responses were used to estimate internal consistency, sampling adequacy, and structural similarity of the instruments across countries (Bilbao et al., 2009).

### 2.3. Psychometric Evaluation

Psychometric analyses included the estimation of Cronbach’s alpha coefficients, Kaiser–Meyer–Olkin (KMO) statistics, Bartlett’s tests of sphericity, exploratory factor analyses, eigenvalues of the first and second factors, and the variance explained by the first factor. Bartlett’s tests were estimated separately within each country and for each scale; therefore, their *p*-values do not represent direct comparisons between Mexico and Russia, but rather within-country assessments of whether the item correlation matrices were appropriate for factor analysis (Rosner, 2010).

To evaluate structural similarity between countries, first-factor loading vectors were compared using Tucker’s coefficient of congruence (φ) and the mean absolute loading difference. Complete item-level details, including corrected item–total correlations (CITC), loading matrices, and item-by-item differences between countries, are provided in the Supplementary Materials (Table S1).

Because the primary objective of the study was to compare psychosocial associations across countries rather than to establish cross-cultural measurement equivalence, formal multi-group confirmatory factor analyses for configural, metric, and scalar invariance were not performed. Consequently, the psychometric analyses were intended to provide evidence of internal consistency and structural similarity rather than formal measurement invariance.

The Russian-language versions were administered by the collaborating investigators at Saint Petersburg State University (for the NOS and Linking People Scale) and taken from published sources (for the Yamagishi and Rosenberg scales). These versions correspond to those routinely used in previous research conducted by the participating group. Although formal published validation studies are not available for all instruments, identical response formats and scoring procedures were applied across language versions. Complete psychometric results are provided in Supplementary Tables S1 and S2.

### 2.4. Statistical Analysis

Descriptive statistics were initially calculated to characterize the sample, including age distribution, sex composition, and study country (Mexico vs. Russia). Distributional properties of the continuous variables were examined descriptively using skewness statistics and visual inspection of quantile–quantile (Q–Q) plots. These procedures were intended to characterize the observed distributions rather than to establish the suitability of the regression models. To reduce sensitivity to heteroskedasticity and potential deviations from classical regression assumptions, the multivariable linear models were estimated using ordinary least squares regression with HC3 robust standard errors (Rosner, 2010).

Psychometric properties of the study scales were evaluated prior to substantive analyses. Internal consistency was assessed using Cronbach’s alpha coefficients. Sampling adequacy for factor analysis was examined using the Kaiser–Meyer–Olkin (KMO) statistic, and Bartlett’s tests of sphericity were used to confirm that the correlation matrices were appropriate for factor analysis. Exploratory factor analyses were conducted to verify the dominant factor structure of each scale within each country.

Mean scale scores were compared between countries using Welch’s *t* tests to account for potential heterogeneity of variances between groups. Pearson correlation coefficients were estimated to examine associations among psychosocial constructs, and differences between correlation coefficients across countries were tested using Fisher’s *r*-to-*z* transformation. Multivariable models were subsequently estimated to evaluate associations between psychosocial factors and network orientation while adjusting for age and sex.

Continuous multivariable interaction models constituted the primary and only inferential analyses. Ordinary least squares regression models with HC3 robust standard errors were used to evaluate the associations between psychosocial variables and negative network orientation while accounting for interaction effects with country. Because dichotomization of continuous variables reduces statistical information and statistical power (MacCallum et al., 2002), the logistic regression analyses included in the previous version of the manuscript were removed from the revised analysis.

All primary statistical analyses were conducted using IBM SPSS Statistics for Windows version 26 (IBM Corp., Armonk, NY, USA) (Dudley et al., 2004; Ho et al., 2011).

## 3. Results

Of the 738 initially screened participants, 20 were excluded for being outside the eligible age range, and 42 were excluded because their country of birth was neither Mexico nor Russia, while no participants were removed due to incomplete questionnaires, resulting in a final sample of 676 university students (Mexico, *n* = 422; Russia, *n* = 254). The Rosenberg Self-Esteem Scale demonstrated the highest reliability among the evaluated instruments. Overall, the study scales showed acceptable internal consistency across both countries, with Cronbach’s α coefficients ranging from 0.70 to 0.90 (Table 1).

The distributions of the continuous variables exhibited skewness values within acceptable ranges for parametric analyses (Table 2). To enhance transparency regarding the psychometric performance of the instruments across countries, supplementary analyses were conducted to evaluate internal consistency, sampling adequacy, and factorial similarity. These analyses included Cronbach’s α coefficients, Kaiser–Meyer–Olkin (KMO) statistics, Bartlett’s tests of sphericity, exploratory factor analyses, Tucker’s coefficient of congruence (φ), and mean absolute loading differences. Tucker’s congruence coefficients indicated a high degree of similarity in the dominant factor structure across countries for all study instruments (Supplementary Tables S1–S5). However, these analyses should be interpreted as evidence of structural similarity rather than formal measurement invariance.

In addition, cross-national factorial congruence metrics, including Tucker’s coefficient of congruence (φ) and mean absolute loading differences, were estimated. Standardized comparisons of scale scores and country-specific correlation matrices among the psychological constructs were also included. Collectively, these analyses provide additional support for the internal consistency, latent structure comparability, and interpretability of the primary cross-national findings (see Supplementary Tables S1–S5).

Distributional checks did not reveal substantial deviations from symmetry in the total scale scores. Absolute skewness values remained well below 2, supporting the appropriateness of using parametric statistical analyses.

### 3.1. Sample Characteristics and Mean Differences Between Countries

The Mexican sample was, on average, slightly older (0.3 years) and included a higher proportion of women compared with the Russian sample (Table 3).

Mean comparisons (Table 4) showed that the Russian sample scored significantly higher on generalized trust and self-esteem, the latter demonstrating a particularly large effect size (Hedges’ g = 1.42). In contrast, NOS scores and interpersonal sympathy did not differ significantly between countries. The absence of cross-national differences in network avoidance (NOS) is particularly noteworthy, as it suggests that the subsequent divergences observed in the relationships between variables cannot be attributed to differences in average levels of network avoidance. Additional descriptive statistics and standardized comparisons are provided in Supplementary Table S3.

### 3.2. Sex-Stratified Comparisons Between Countries

To evaluate whether the observed differences could be attributed to sex composition, psychosocial scale scores were compared between countries within each sex stratum (Table 5). The main patterns remained consistent across analyses: self-esteem was significantly higher in the Russian sample among both men and women, whereas NOS scores did not differ significantly between countries in either sex stratum.

These sex-stratified comparisons demonstrate that the cross-cultural difference in self-esteem remained statistically significant among both men and women despite the corrected RSES scoring, indicating that the country effect cannot be explained by differences in sex composition. Similarly, generalized trust remained consistently higher in the Russian sample across both strata. In contrast, network orientation showed only minimal differences within each sex group, reinforcing the finding that overall levels of orientation toward help-seeking are comparable between countries.

However, a consistent pattern emerged for network orientation. In both Mexico and Russia, women reported significantly lower NOS scores than men, indicating a somewhat lower network avoidance of social networks (see Table 6). The magnitude of this difference was small but consistent across countries, corresponding to Cohen’s d = 0.21 in Mexico and d = 0.31 in Russia. No significant sex differences were observed for self-esteem or generalized trust in either country. Interpersonal sympathy also did not differ significantly between men and women in the Mexican sample. In contrast, Russian men reported significantly higher levels of interpersonal sympathy than Russian women.

### 3.3. Correlational Structure of Psychosocial Variables

The correlation matrix derived from the combined data from both countries showed that higher generalized trust was associated with lower negative network orientation, whereas greater interpersonal sympathy was associated with higher network avoidance. The zero-order correlation between self-esteem and NOS was nearly null in the combined sample; however, this aggregate pattern concealed markedly divergent national trends (Table 7).

Comparisons between the correlation coefficients observed in Mexico and Russia using Fisher’s r-to-z tests are presented in Table 8. Two findings were particularly noteworthy. First, self-esteem showed no significant correlation with network orientation in the Mexican sample (r = 0.04), whereas a significant inverse correlation was observed in the Russian sample (r = −0.286). The difference between countries remained statistically significant (Fisher’s Z = 4.18, *p* < 0.001). Second, generalized trust demonstrated a significantly stronger negative correlation with network avoidance (NOS) in the Russian sample than in the Mexican sample (*p* = 0.023). In contrast, interpersonal sympathy showed a positive correlation with NOS in both countries, with no significant differences between them (*p* = 0.521). The complete country-specific correlation matrices are presented in Supplementary Tables S4 and S5.

### 3.4. Multivariable Continuous Models

To evaluate whether participants’ country of origin influenced network orientation after simultaneous adjustment for other psychosocial factors, age, and sex, a robust linear regression model was estimated using continuous NOS scores as the outcome variable and predictor × country interaction terms. Three main findings emerged from the adjusted continuous model presented in Table 9.

To further examine the country-specific simple slopes derived from the interaction model, additional analyses were conducted. In Mexico, self-esteem was not significantly associated with network orientation (B = 0.120, *p* = 0.403). In contrast, in Russia, higher self-esteem was significantly associated with lower network orientation (B = −0.883, *p* < 0.001) (see Table 10).

Interpersonal sympathy remained positively associated with network orientation in both countries, whereas generalized trust remained negatively associated with network orientation, with a significantly stronger association in Russia. Specifically, each one-point increase in generalized trust was associated with an approximate reduction of 0.48 points in NOS in Mexico and nearly 0.99 points in NOS in Russia. This pattern indicates a substantially stronger trust–help-seeking gradient in the Russian subsample.

## 4. Discussion

The present study demonstrates that similar average levels of network avoidance in Mexico and Russia do not imply an equivalent psychosocial architecture. Although mean NOS scores were comparable across countries, the configuration of their associations differed substantially, confirming that intercultural comparisons based solely on average scores may obscure important differences in the psychological associations underlying help-seeking behavior. This finding is particularly relevant because it suggests that two populations may arrive at similar levels of social network avoidance through distinct psychosocial pathways, with different implications for intervention, prevention, and theoretical interpretation. The continuous multivariable interaction models consistently demonstrated that the association between self-esteem and negative network orientation differed significantly between Mexico and Russia after adjustment for generalized trust, interpersonal sympathy, age, and sex.

One possible interpretation of this pattern is that the psychological meaning and behavioral implications of self-esteem may differ across sociocultural contexts. Rather than functioning as a universal facilitator or inhibitor of help-seeking, self-esteem may be associated with different interpersonal attitudes and expectations depending on the social environment in which it is expressed. This interpretation is consistent with evidence indicating that the psychological meaning and social functions of positive self-evaluation vary across cultural contexts, influencing how self-esteem is expressed and translated into interpersonal behavior (Becker et al., 2014). Likewise, previous studies have suggested that the relationship between self-esteem and help-seeking may vary according to the broader social context in which interpersonal relationships are embedded (Hofstede et al., 2010; Ostrom, 1990). However, because the present study did not directly assess constructs such as self-sufficiency, competence norms, honor, or concerns about vulnerability, these interpretations should be regarded as possible contextual explanations rather than mechanisms supported by the current data. Furthermore, the cross-sectional design does not permit causal inferences; therefore, future studies incorporating direct measures of these constructs are needed to clarify the processes underlying the observed cross-national differences.

Generalized trust displayed a relatively consistent pattern across countries. In both national samples, greater trust was associated with lower network avoidance, supporting the notion that trusting others reduces the perceived interpersonal risk associated with seeking support (Luhmann et al., 2017; Yamagishi, 2019). Nevertheless, this association was significantly stronger in Russia than in Mexico, suggesting that the role of trust in facilitating help-seeking may vary across contexts.

Interpersonal sympathy produced the most counterintuitive, yet also one of the most stable, findings: in both countries, higher levels of sympathy were associated with greater network avoidance. Rather than representing a contradiction, this pattern may reflect a form of interpersonal sensitivity that heightens concern about burdening, disturbing, or inconveniencing others when requesting help. In other words, individuals with greater sympathy may be precisely those who internalize the relational cost of support-seeking more strongly, thereby becoming less likely to mobilize their social networks even when they recognize the need for assistance. The consistency of this effect across Mexico and Russia suggests that this association may represent a relatively stable cross-cultural phenomenon, less dependent on national context than the relationship observed for self-esteem. Similarly, cultural differences in the motivations underlying support-seeking have been shown to influence both the decision to seek support and the emotional consequences of receiving it, emphasizing that help-seeking behaviors are shaped by culturally embedded interpersonal goals (Ishii et al., 2017).

Sex differences also provided important nuances. In both countries, men exhibited greater negative network orientation than women, indicating a somewhat lower willingness to seek social support. This pattern is consistent with the literature on gender and interpersonal help-seeking, which often reports greater female openness toward relational coping and emotional disclosure (Lippa, 2010). An unexpected finding was the absence of self-esteem differences between men and women in both national samples. This finding contrasts with the study by Bleidorn et al. (2016), conducted across 48 cultures with nearly one million respondents, in which men consistently exhibited higher global levels of self-esteem. Although the corrected analyses substantially reduced the magnitude of the between-country difference, the principal pattern of association between self-esteem and network orientation remained unchanged.

Given that the Single-Item Self-Esteem Scale (SISE) used in that study and the Rosenberg Self-Esteem Scale (RSES) employed in our study have demonstrated strong convergent validity, the divergence in findings is unlikely to be attributable solely to differences in the measurement instruments (Robins et al., 2001). In our data, however, sex differences were relatively small and did not account for the primary cross-cultural effects, since the country × self-esteem interaction remained significant among both men and women. This suggests that the observed variations are not merely a byproduct of demographic composition, but rather reflect a deeper structural relationship between the psychosocial constructs examined.

Although the revised analyses substantially reduced the magnitude of the between-country difference in self-esteem after correcting the original scoring error, the observed effect remained relatively large compared with previous international studies. Therefore, this finding should be interpreted cautiously. Besides reflecting characteristics specific to the participating university samples, differences in response styles or other forms of cross-cultural measurement variability remain plausible alternative explanations that could not be evaluated within the present study. Future studies incorporating formal tests of measurement invariance and analyses of response styles will be important to determine whether the observed differences represent true cross-cultural variation or are partially influenced by the psychometric characteristics of the instruments.

Geert Hofstede’s cultural framework offers a useful lens through which to contextualize these findings, provided that it is applied cautiously and not treated as a deterministic explanation (Hofstede et al., 2010). Because individual-level cultural orientation was not directly measured, the frameworks proposed by Hofstede, Hall, and Schwartz are used solely as conceptual perspectives to contextualize the findings rather than as empirical explanations of the observed associations (Hall, 2003; Hofstede et al., 2010; Schwartz, 1992). Accordingly, country (Mexico vs. Russia) should be interpreted as a contextual variable encompassing multiple social, historical, educational, institutional, and cultural influences (Minkov et al., 2022) rather than as a direct measure of culture. Therefore, the mechanisms underlying the observed cross-national differences remain hypothetical and should be directly evaluated in future studies using validated measures of cultural orientation. Within this framework, the differing psychosocial configurations observed despite similar average network orientation scores may reflect contextual differences in how self-esteem, trust, and help-seeking are socially interpreted.

Beyond cultural and psychological factors, ecological and socio-environmental conditions may also influence help-seeking behaviors and network orientation. Characteristics such as climate, opportunities for outdoor social interaction, urban design, and the availability of public spaces may shape the development and maintenance of social support networks. However, because these contextual factors were not assessed in the present study, they are discussed only as potential contextual influences rather than empirical explanations of the observed associations (Jennings & Bamkole, 2019; Maas et al., 2009; Oishi & Graham, 2010; Van Lange et al., 2017). Future cross-national research integrating psychosocial, cultural, and environmental variables is warranted to clarify the contribution of these factors to differences in network orientation (Kawachi & Berkman, 2023).

In this sense, the central contribution of the present study is not to demonstrate that one country is inherently “more” or “less” likely to seek help, but rather to show that equivalent mean scores may coexist with different observed psychosocial association patterns. However, because formal measurement invariance was not evaluated, these findings should be interpreted as evidence of sample-specific associative structures rather than definitive cross-cultural differences (Berry et al., 2011; Heine, 2020; Schwartz, 1992). Consequently, the present findings should be regarded as hypothesis-generating rather than definitive evidence of cross-cultural differences. This distinction is theoretically important because it encourages moving beyond superficial comparisons of average scores toward analyses focused on relationships among variables, which are more sensitive to the underlying mechanisms of psychological functioning (Berry et al., 2011; Lonner & Berry, n.d.; Schwartz, 1992). For comparative mental health research, this represents a particularly valuable conclusion, as interventions designed solely from national averages may prove insufficient if identical scores carry different meanings across cultural contexts (Heine, 2020; Oishi & Graham, 2010).

Nevertheless, several limitations should be acknowledged. The cross-sectional design precludes establishing causal directionality and does not allow determination of whether self-esteem, trust, or interpersonal sympathy precedes network orientation or vice versa. Furthermore, all measures relied on self-report instruments and may therefore be influenced by social desirability, response styles, or other unmeasured contextual factors. The sample was restricted to university students, limiting the generalizability of the findings to other populations. In addition, although the analysis compared two countries, individual-level cultural dimensions such as individualism–collectivism, communication context, and personal value orientations were not directly measured. Consequently, country (Mexico vs. Russia) should be interpreted as a contextual comparison variable rather than a direct measure of culture, and the theoretical perspectives of Hofstede, Hall, and Schwartz should be regarded as conceptual frameworks rather than empirically tested mechanisms. Future studies incorporating validated measures of cultural orientation are needed to clarify the mechanisms underlying the observed cross-national differences. Additionally, participants were recruited from multiple universities within each country; however, clustering by institution was not modeled. Therefore, residual within-university correlation cannot be completely excluded.

An additional finding deserving attention was the opposite direction of the association between generalized trust and self-esteem across countries. Although this correlation was modest in magnitude, it suggests that self-esteem may be embedded within different psychosocial contexts in the two university samples. Because formal measurement invariance and individual-level cultural orientations were not directly evaluated, this finding should be interpreted cautiously and regarded as hypothesis-generating rather than definitive evidence of cross-cultural differences. Future studies should examine whether this pattern is replicated using latent variable approaches and direct measures of cultural orientation.

Although the study demonstrated acceptable internal consistency, factorability, and structural similarity of the study instruments across countries, formal tests of measurement invariance (e.g., configural, metric, and scalar invariance) were not performed. Furthermore, formal published validation studies were not available in the moment of the study for every language version of the instruments used, particularly for Russian and Spanish versions of the Network Orientation Scale and the Linking People Scale. Consequently, cross-national comparisons should be interpreted with appropriate caution, as some observed differences may partially reflect cross-cultural measurement characteristics rather than exclusively true differences in the underlying psychological constructs. Future studies should formally evaluate measurement invariance across languages and populations to strengthen the validity of cross-cultural comparisons.

In addition, information regarding participants’ academic disciplines and socioeconomic characteristics was not collected uniformly across the participating institutions and therefore could not be incorporated into the analyses. Consequently, residual compositional differences between the two convenience samples can not be excluded and may have contributed, at least in part, to the observed cross-national differences. Finally, data were collected during 2023–2024, a period characterized by dynamic social and geopolitical circumstances that may have differentially influenced university students, particularly in Russia. Consequently, period-specific contextual influences cannot be disentangled from broader cross-national differences within the present study design.

Accordingly, the present findings should be interpreted as differences in observed psychosocial association patterns within the participating university samples rather than definitive evidence of cross-cultural differences in the underlying psychological constructs. Rather than representing definitive cross-cultural differences, the observed associations should be interpreted as hypothesis-generating evidence requiring confirmation in future studies incorporating formal measurement invariance, latent variable approaches, and broader cross-cultural samples. Future studies incorporating formal measurement invariance and latent variable approaches will be necessary to determine whether these patterns reflect true cross-cultural variation.

## 5. Conclusions

In conclusion, among university students from Mexico and Russia, network avoidance (NOS) showed similar average levels but different observed psychosocial association patterns. Self-esteem emerged as the most context-dependent association, generalized trust functioned as a protective factor in both countries with a stronger effect in Russia, and interpersonal sympathy remained a stable correlate of network avoidance. Sex differences were consistent but small and did not alter the principal pattern of the study. Collectively, these findings suggest that willingness to seek social support should not be interpreted solely through comparisons of mean scores between populations, but also through the psychosocial associations underlying these dispositions within each contextual setting. Continuous multivariable interaction analyses demonstrated that the association between self-esteem and negative network orientation differed significantly between the Mexican and Russian university samples, whereas generalized trust and interpersonal sympathy showed relatively stable associations across both samples. Because formal measurement invariance and individual-level cultural dimensions were not directly evaluated, these findings should be interpreted as hypothesis-generating rather than definitive evidence of cross-cultural differences. Future studies incorporating formal measurement invariance, latent variable approaches, and direct measures of cultural orientation will be necessary to confirm and extend these observations.

## Figures and Tables

**Table 1:** Psychometric Properties of the Study Instruments.

			Cronbach’s Alpha Coefficients		
Scale	Items	N	Total	Mexico	Russia
Generalized Trust Scale	6	676	0.79	0.80	0.77
Network Orientation Scale	20	676	0.73	0.70	0.81
Rosenberg Self-Esteem Scale	10	676	0.90	0.84	0.82
Linking People Scale (LPS)	15	676	0.80	0.78	0.84

Note: Cronbach’s alpha coefficients are presented for the complete analytical sample (both countries combined) and separately for Mexico and Russia. Reliability estimates were calculated using the final analytical dataset employed in all substantive models. Higher alpha values indicate greater internal consistency of item responses within each scale.

**Table 2:** Descriptive Statistics and Distributional Properties of Continuous Variables.

Variable	N	Mean	SD	Min	Max	Median	Skewness
Generalized trust	676	17.97	4.16	6	30	18	−0.09
Network orientation (NOS)	676	51.95	8.55	25	83	52	0.35
Self-esteem	676	24.99	2.99	13	34	25	−0.16
Linking People Scale (LPS)	676	39.38	8.91	17	68	39	0.21

Note: Skewness was examined for each continuous total score in the combined sample. Values close to zero indicate approximate symmetry, and all observed skewness values were small in magnitude. These distributional diagnostics support the use of parametric comparisons and regression models for the scale totals.

**Table 3:** Demographic Characteristics by Country.

Characteristic	Mexico (*n* = 422)	Russia (*n* = 254)	Test Statistic	*p*
Age, mean (SD)	20.32 (1.84)	20.01 (1.93)	Welch’s *t* = 2.01 (df = 513.80)	0.044
Women, *n* (%)	286 (67.8%)	149 (58.7%)	χ^2^ = 5.35 (df = 1)	0.021
Men, *n* (%)	136 (32.2%)	105 (41.3%)		

Age was compared using Welch’s *t* test for unequal variances. Sex distribution was compared using Pearson’s χ^2^ test. Percentages represent column percentages within each country.

**Table 4:** Cross-National Comparisons of Psychosocial Scale Scores.

Variable	Mexico Mean (SD)	Russia Mean (SD)	Welch’s *t*	*p*	Hedges’ g
Generalized trust	17.38 (4.11)	18.95 (4.07)	−4.85	<0.001	−0.38
Network orientation (NOS)	52.21 (7.72)	51.51 (9.79)	0.96	0.335	0.08
Self-esteem	23.67 (2.50)	27.18 (2.39)	−18.16	<0.001	−1.42
Interpersonal sympathy	39.56 (8.28)	39.08 (9.87)	0.64	0.521	0.05

Note: Values are presented as means (SD) unless otherwise indicated. Welch’s *t* tests were used because they remain valid under unequal variances and unequal group sizes. Hedges’ g is reported as the standardized mean difference (Mexico minus Russia); positive values indicate higher scores in the Mexican sample.

**Table 5:** Sex-Stratified Cross-Cultural Comparisons of Psychosocial Scale Scores.

Sex	Variable	Mexico	Russia	Welch’s *t* (df)	*p*	Hedges’ g
Men	Generalized trust	17.17	18.52	−2.51 (df = 235.67)	0.013	−0.32
Men	Network orientation	53.30	53.25	0.05 (df = 191.77)	0.962	0.01
Men	Self-esteem	23.43	27.30	−12.41 (df = 227.29)	<0.001	−1.60
Men	Interpersonal sympathy	39.86	41.04	−0.98 (df = 202.68)	0.330	−0.13
Women	Generalized trust	17.48	19.26	−4.29 (df = 284.17)	<0.001	−0.44
Women	Network orientation	51.69	50.29	1.52 (df = 248.45)	0.130	0.16
Women	Self-esteem	23.79	27.09	−13.36 (df = 312.96)	<0.001	−1.33
Women	Interpersonal sympathy	39.41	37.70	1.84 (df = 262.98)	0.067	0.20

Values correspond to cross-national comparisons within each sex stratum. Welch’s *t* tests were used because unequal variances were assumed. Hedges’ g is reported as the standardized mean difference (Mexico minus Russia).

**Table 6:** Sex Differences in Psychosocial Variables within Each Country.

Variable	Mexico Men Mean (SD)	Mexico Women Mean (SD)	Welch’s *t* (df)	*p*	Hedges’ g	Russia Men Mean (SD)	Russia Women Mean (SD)	Welch’s *t* (df)	*p*	Hedges’ g
Network orientation (NOS)	53.30 (7.52)	51.69 (7.76)	2.04 (df = 273.13)	0.042	0.21	53.25 (9.66)	50.29 (9.72)	2.40 (df = 224.87)	0.017	0.30
Self-esteem	23.43 (2.45)	23.79 (2.52)	−1.40 (df = 272.49)	0.163	−0.14	27.30 (2.37)	27.09 (2.40)	0.72 (df = 226.03)	0.475	0.09
Generalized trust	17.17 (4.47)	17.48 (3.93)	−0.70 (df = 236.86)	0.485	−0.08	18.52 (3.89)	19.26 (4.18)	−1.43 (df = 233.58)	0.154	−0.18
Interpersonal sympathy	39.86 (8.38)	39.41 (8.25)	0.52 (df = 261.71)	0.607	0.05	41.04 (9.93)	37.70 (9.62)	2.67 (df = 219.66)	0.008	0.34

Higher scores on the Network Orientation Scale indicate greater reluctance to seek support from social networks. Hedges’ g is reported as the standardized mean difference (men minus women); positive values indicate higher scores among men.

**Table 7:** Pearson Correlations among Psychosocial Variables in the Total Sample and by Country.

Variable	1	2	3	4
Total Sample (N = 676)				
1. Generalized trust	—			
2. Network orientation (NOS)	−0.411 ***	—		
3. Self-esteem RSES	0.117 **	−0.101 **	—	
4. Interpersonal sympathy	−0.217 ***	0.389 ***	−0.039	—
Mexico (*n* = 422)				
1. Generalized trust	—			
2. Network orientation (NOS)	−0.350 ***	—		
3. Self-esteem RSES	−0.064	0.04	—	
4. Interpersonal sympathy	−0.217 ***	0.409 ***	−0.026	—
Russia (*n* = 254)				
1. Generalized trust	—			
2. Network orientation (NOS)	−0.498 ***	—		
3. Self-esteem RSES	0.156 *	−0.286 ***	—	
4. Interpersonal sympathy	−0.216 ***	0.366 ***	−0.036	—

NOS = Network Orientation Scale. Higher NOS scores indicate greater reluctance to seek support from social networks. * *p* < 0.05; ** *p* < 0.01; *** *p* < 0.001.

**Table 8:** Fisher’s r-to-z Tests Comparing Correlations between Mexico and Russia.

Compared Variables	r Mexico	r Russia	Z	*p*
Generalized trust—Network orientation	−0.350	−0.498	2.27	0.023
Self-esteem—Network orientation	0.04	−0.286	4.18	<0.001
Interpersonal sympathy—Network orientation	0.409	0.366	0.64	0.521
Generalized trust—Self-esteem	−0.064	0.156	−2.78	0.005

Note: Fisher’s r-to-z transformations were used to test whether independent correlation coefficients differed between the Mexican and Russian subsamples. Significant *p*-values indicate that the strength or direction of a given association was not equivalent across countries.

**Table 9:** Multivariable Linear Model for Continuous Network Orientation.

Predictor	B	95% CI	*p*
Intercept	53.01	[51.91, 54.10]	<0.001
Self-esteem (centered)	0.12	[−0.162, 0.403]	0.403
Interpersonal sympathy (centered)	0.328	[0.233, 0.423]	<0.001
Generalized trust (centered)	−0.502	[−0.668, −0.335]	<0.001
Country (Russia vs Mexico)	2.358	[0.733, 3.983]	0.004
Self-esteem × Country	−1.003	[−1.571, −0.434]	0.001
Interpersonal sympathy × Country	−0.074	[−0.224, 0.075]	0.331
Generalized trust × Country	−0.468	[−0.789, −0.146]	0.004
Age (centered)	0.116	[−0.196, 0.427]	0.466
Sex (Women vs Men)	−1.490	[−2.660, −0.321]	0.012

Ordinary least squares model with HC3 robust standard errors. The dependent variable was the continuous NOS total score, where higher values indicate greater network avoidance. Continuous predictors were mean-centered; therefore, the country coefficient estimates the Mexico–Russia difference at the pooled mean of the psychosocial scores. Model fit: R^2^ = 0.308, adjusted R^2^ = 0.299, N = 676.

**Table 10:** Country-Specific Simple Slopes Derived from the Interaction Model.

Predictor	Mexico B (95% CI)	*p*	Russia B (95% CI)	*p*
Self-esteem	0.120 [−0.162, 0.403]	0.403	−0.883 [−1.373, −0.392]	<0.001
Interpersonal sympathy	0.328 [0.233, 0.423]	<0.001	0.253 [0.139, 0.368]	<0.001
Generalized trust	−0.502 [−0.668, −0.335]	<0.001	−0.969 [−1.245, −0.693]	<0.001

Note: Simple slopes were derived from linear combinations of the main-effect and interaction terms presented in Table 9. The Mexican slope corresponds to the main effect of each centered predictor, whereas the Russian slope equals the main effect plus the corresponding predictor × country interaction term. These estimates represent the adjusted association between each psychosocial variable and the continuous NOS outcome within each country.

## Data Availability

The anonymized dataset and the statistical analysis syntax supporting the findings of this study are available from the corresponding author upon reasonable request. Access to these materials will be granted in accordance with the collaborative agreements established among the participating institutions in Mexico and Russia and applicable ethical and institutional requirements.

## Supplementary Materials

The following supporting information can be downloaded at https://www.mdpi.com/article/10.3390/bs16081299/s1, Supplementary Questionnaire: Complete wording, response options, and scoring procedures for the Network Orientation Scale (NOS), Rosenberg Self-Esteem Scale (RSES), Liking People Scale (LPS), and the 6-item Yamagishi Generalized Trust Scale in English, Spanish, and Russian. Table S1: Internal consistency and factorability of the study scales by country. Table S2: Cross-national factorial congruence between Mexico and Russia. Table S3: Means, standard deviations, and cross-national comparisons of scale scores. Table S4: Correlation matrix between psychological scales in Mexico. Table S5: Correlation matrix between psychological scales in Russia. Reference (Arbuzova & Gorbatov, 2021) is cited in the Supplementary Materials.
