# Supplementary material for: Psychosocial Correlates of Network Orientation: A Cross-National Comparison of University Students in Mexico and Russia
Source: Behav Sci (Basel). 2026 Jul 30;16(8):1299. doi: 10.3390/bs16081299 (PMC13509760; doi:10.3390/bs16081299)
Supplement: Supplementary file 1 [file behavsci-16-01299-s001.zip › behavsci-4383536-supplementary.pdf]

Supplementary Materials

Network Orientation Scale by Vaux A., Burda P. & Stewart D.B. (20 items)

The NOS consists of 20 items.

Each ítem is answered on 4-point Likert scale:
1- Strongly agree
2-Agree
3- Disagree
4- Strongly disagree

Directly scored items ("Strongly agree" is equal to one point): 1, 2, 4, 6, 7, 9, 11, 14, 16, 19
Revers-scored items ("Strongly agree" is equal to four points): 3, 5, 8, 10, 12, 13, 15, 17, 18. 20

The total score is obtained by summing the items after recording, those worded negatively when the goal is to obtain a score indicating a positive orientation toward social support.

Vaux, A., Burda, P., & Stewart, D. (1986). Orientation toward utilization of support resources. *Journal of Community Psychology*, *14*(2), 159–170. https://doi.org/10.1002/1520-6629(198604)14:2<159::AID-JCOP2290140207>3.0.CO;2-H

Spanish version:
Items:

1. A veces es indispensable hablar con alguien sobre mis problemas
2. Amigos a menudo pueden dar un buen consejo
3. Debes tener cuidado con aquellos a quienes le cuentas tus secretos,
4. A menudo aprendo información útil de otras personas,
5. La gente debería guardar sus problemas para ellos mismos,
6. Es fácil para mí hablar de asuntos personales con otros,
7. En el pasado, los amigos realmente me ayudaban cuando tenía problemas,
8. Nunca puedes confiar en la gente con tus secretos,
9. Cuando una persona está molesta, necesita hablar con un amigo,
10. Otras personas nunca entienden mis problemas,
11. Casi todo el mundo conoce a alguien en quien puede confiar con un secreto,

12. Si no puedes resolver tus problemas, nadie puede ayudarte,
13. En el pasado, cuando tenía problemas, pensaba que las opiniones de otras personas eran inútiles,
14. Cuando estás enojado, te hace sentir mejor decirle a un amigo lo que pasó
15. Algunas cosas son demasiado personales para hablar con nadie,
16. Es muy fácil para mí decir en quién puedo confiar y en quién no,
17. En el pasado, me han intimidado personas en las que confiaba,
18. Si usted confía en otras personas, ellas lo usarán,
19. Creo que es normal pedir un favor a la gente,
20. Incluso si necesito algo, no me atrevo a pedirlo,

Response options:

1. Totalmente de acuerdo
2. De acuerdo
3. En desacuerdo
4. Totalmente en desacuerdo

Translated and validated for Spanish by our group

Russian Version:

1. Иногда необходимо поговорить с кем-то о своих проблемах,
2. Друзья часто могут дать хороший совет,
3. Вы должны быть осторожны с теми, кому рассказываете свои секреты,
4. Я часто узнаю полезную информацию от других людей,
5. Люди должны держать свои проблемы при себе,
6. Мне легко говорить о личных вопросах с другими,
7. В прошлом друзья действительно помогали мне, когда у меня возникали проблемы,
8. Никогда нельзя доверять людям свои секреты,
9. Когда человек расстроен, ему необходимо поговорить с другом,
10. Другие люди никогда не понимают моих проблем,
11. Почти каждый человек знает кого-то, кому он может доверить секрет,
12. Если вы не сможете разобраться в своих проблемах – вам в этом никто не сможет помочь,
13. В прошлом, когда у меня были проблемы, я считал мнение других людей бесполезным,
14. Когда вы сердитесь, вам становится лучше, если вы рассказываете другу о том, что произошло,
15. Некоторые вещи слишком личные, чтобы говорить о них с кем- либо,
16. Мне довольно легко определить, кому можно доверять, а кому нельзя,

17. В прошлом меня обижали люди, которым я доверял,
18. Если ты доверишься другим людям, они воспользуются тобой,
19. Я считаю нормальным просить людей об одолжении,
20. Даже если мне что-то нужно, я не решаюсь попросить это,

Response options:
1. Абсолютно согласен
2. Согласен
3. Не согласен
4. Абсолютно не согласен

Arbuzova, E. N., & Gorbatov, S. V. (2025). *Adaptation and initial validation of methods for studying affiliative characteristics of personality*. En *Education, Innovations, Research as a Resource for Community Development: Proceedings of the All-Russian Scientific-Practical Conference with International Participation* (Cheboksary, Russia, March 26, 2025). ID "Sreda". https://doi.org/10.31483/a-10718

English version:
1. Sometimes it is necessary to talk to someone about your problems
2. Friends often have good advice to give
3. I often get useful information from (other people) others
4. People should keep their problems to themselves
5. It's easy for me to talk about personal and private matters
6. In the past, friends have really helped me out when I've had a problem
7. You can never trust people to keep a secret
8. When a person gets upset they should talk it over with a friend
9. Other people never understand my problems
10. Almost everyone knows someone they can trust with a personal secret
11. If you can't figure out your problems, nobody can
12. In the past, I have rarely found other people's opinions helpful when I have aproblem
13. It really helps when you are angry to tell a friend what happened
14. Some things are too personal to talk to anyone about
15. If you confide in other people, they will take advantage of you
16. It's ok to ask favors of people
17. Even if I need something, I would hesitate to borrow it from someone
18. You have to be careful who you tell personal things to
19. I think it is normal to ask people for favors.
20. Even if I need something, I do not dare to ask for it.

Response options:

1. Strongly agree
2. Agree
3. Disagree
4. Strongly disagree

Linking People Scale (LPS) by Filsinger E.E. (15 items)

the scale consists of 15 Likert-type items;
the score is obtained by summing the items;
the original article does not present interpretive categories ("high," "medium," "low").

Filsinger, E. E. (1981). A Measure of Interpersonal Orientation: The Linking People Scale. *Journal of Personality Assessment*, *45*(3), 295–300. https://doi.org/10.1207/S15327752JPA4503_11

Spanish version:

1. A veces cuando la gente me habla, espero en mi corazón que se vayan pronto,
2. Mi necesidad de gente es bastante baja,
3. Uno de los problemas de las personas modernas es que dependen demasiado unas de otras,
4. Mis experiencias más felices están relacionadas con otras personas,
5. Para mi felicidad personal, realmente no necesito a la gente,
6. El propio carácter se desarrolla sólo en el flujo de los acontecimientos de la vida,
7. Podría ser feliz viviendo lejos de la gente,
8. Es importante para mí llevarme bien con otras personas,
9. Cualquier cosa que haga, preferiría hacerlo en compañía de otras personas,
10. Sin duda - Me encanta la gente,
11. El carácter de una persona se desarrolla en la soledad (independientemente),
12. En general, no me gusta la gente,
13. Excepto por mis amigos cercanos, no me gusta la gente,
14. El tiempo siempre es corto, y otras personas están perdiendo mi tiempo,
15. La gente es lo más importante en mi vida,

Response options:

1. Totalmente de acuerdo

2. De acuerdo
3. No estoy seguro
4. En desacuerdo
5. Totalmente en desacuerdo

Translated to Spanish and validated by our group

Russian version:

1. Иногда, когда люди разговаривают со мной, в душе я надеюсь, что они поскорее уйдут,
2. Моя потребность в людях довольно низкая,
3. Одна из проблем современных людей – это то, что они слишком зависят друг от друга,
4. Мои самые счастливые переживания связаны с другими людьми,
5. Для моего личного счастья люди не особо нужны,
6. Собственный характер человека развивается только в потоке жизненных событий,
7. Я мог бы быть счастлив, живя вдали от людей,
8. Для меня важно уметь ладить с другими людьми,
9. Что бы я ни делал, я предпочел бы делать это в компании других людей,
10. Нет сомнений – я люблю людей,
11. Свой характер человек вырабатывает в уединении (самостоятельно),
12. В целом, люди мне не нравятся,
13. За исключением моих близких друзей, люди мне не нравятся,
14. Времени и так всегда не хватает, а другие люди только тратят моё время зря,
15. Люди – это самое важное, что есть в моей жизни,

Response options:

1. Совершенно согласен
2. Согласен
3. Не уверен
4. Не согласен
5. Совершенно не согласен

Arbuzova, E. N., & Gorbatov, S. V. (2025). Adaptation and initial validation of methods for studying affiliative characteristics of personality. *Education, Innovations, Research as a Resource for Community Development: Proceedings of the All-Russian Scientific-Practical Conference with International Participation* (Cheboksary, Russia, March 26, 2025). ID "Sreda". https://doi.org/10.31483/a-10718

Arbuzova, E. N., & Gorbatov, S. V. (2021). Psychometric properties of the Russian version of the Liking People Scale. *Current Psychology.*
https://doi.org/10.1007/s12144-019-00435-2

English version:

1. Sometimes when people are talking to me, I find myself wishing that they would leave.
2. My need for people is quite low.
3. One of the things wrong with people today is that they are too dependent upon other people.
4. My happiest experiences involve other people.
5. People are not important for my personal happiness.
6. Personal character is developed in the stream of life.
7. I could be happy living away from people.
8. It is important to me to be able to get along with other people.
9. No matter what I am doing, I would rather do it in the company of other people.
10. There is no question about it - I like people.
11. Personal character is developed in solitude.
12. In general, I don‟t like people.
13. Except for my close friends, I don‟t like people.
14. A person only has a limited amount of time and people tend to cut into it.
15. People are the most important thing in my life.

Response options:

1. Strongly Agree
2. Moderately Agree
3. Neutral
4. Moderately Disagree
5. Strongly Disagree

General Trust Scale by Yamagishi T. & Yamagishi M. (6 items)

• the score is obtained by summing the responses;

• interpretation is done by comparing the scores to the sample distribution (mean, standard deviation, or percentiles).

There are no official categories of:

• Low

• Medium

• High

Yamagishi, T., & Yamagishi, M. (1994). Trust and commitment in the United States and Japan. *Motivation and Emotion*, *18*, 129–166. https://doi.org/10.1007/BF02249397

Spanish version:
All six items in the Russian validation exhibited substantial factor loadings, confirming their psychometric adequacy. To ensure structural equivalence between both instruments, the item missing from the Spanish version—"Most people will respond in kind when they are trusted by others"—was translated from the Russian variant. This process included back-translation verification and semantic proximity comparisons performed by three independent Large Language Models (LLMs: ChatGPT, Gemini, and DeepSeek), yielding a 96.8 % global similarity index. Consequently, both 6-item scales were analyzed using identical scoring procedures, ensuring equivalent and comparable score metrics across both cultural groups.

1. Mayoria de la gente son honestos en general
2. Mayoría de la gente merecen confianza
3. La mayoría de las personas, cuando se les confía, responden al mismo
4. Mayoria de la gente en general son buenos y bondadosos
5. Mayoria de la gente confien en los demás
6. En general confio en los demás

Response options:

1. Totalmente de acuerdo
2. De acuerdo
3. No estoy seguro
4. En desacuerdo
5. Totalmente en desacuerdo

Montoro, A., Shih, P. C., Román, M., & Martínez-Molina, A. (2014). Spanish adaptation of Yamagishi general trust scale. *Anales de Psicología,* 30(1), 302-307.

Russian version:

1. Большинство людей в основном честны
2. Большинство людей заслуживают доверия
3. Большинство людей, когда им доверяют, отвечают тем же
4. Большинство людей в основном хорошие и добрые
5. Большинство людей доверяют другим
6. В общем, я доверяю другим людям

Response options:

1. Совершенно согласен
2. Согласен
3. Не уверен
4. Не согласен
5. Совершенно не согласен

Vlasenko, E. A. (2022). Adaptation of the Russian-language version of T. Yamagishi's Generalized Trust Scale on a sample of students. *Psychology in Education*, 4(4), 509-520. https://doi.org/10.33910/2686-9527-2022-4-4-509-520).

English version:

1. Most people are basically honest

2. Most people are trustworthy
3. Most people trust a person if the person trusts them
4. Most people are basically good-natured and kind
5. Most people trust others
6. Generally, I trust others

Response options:

1. Strongly agree
2. Agree
3. Not sure
4. Disagree
5. Strongly disagree

Self Esteem Scale (RSE) by Rosenberg M. (10 items)

To score the items, assign a value to each of the 10 items as follows:

• For items 1,2,4,6,7: Strongly Agree=4, Agree=3, Disagree=2, and Strongly Disagree=1.

• For items 3,5,8,9,10 (which are reversed in valence, and noted with the asterisks** below): Strongly Agree=1, Agree=2, Disagree=3, and Strongly Disagree=4.

The scale ranges from 0-40, with 40 indicating the highest score possible.

Rosenberg, M. (1965). *Society and the adolescent self-image*. Princeton University Press

Spanish version:

1. En general estoy satisfecho conmigo mismo
2. A veces siento que no soy lo suficientemente bueno
3. Creo que tengo varias ventajas,
4. Muchas cosas puedo hacer igual de bien que la mayoría de las personas
5. No creo que tenga nada de lo que estar orgulloso,
6. A veces me siento inútil

7. Me considero digno e igual a otros seres humanos,
8. Ojalá tuviera más respeto por mí mismo,
9. Básicamente, me considero un perdedor,
10. Estoy bien conmigo mismo,

Response options:

1. Totalmente de acuerdo
2. De acuerdo
3. En desacuerdo
4. Totalmente en desacuerdo

Martín-Albo, J., Núñez, J. L., Navarro, J. G., & Grijalvo, F. (2007). The Rosenberg Self-Esteem Scale: translation and validation in university students. *The Spanish Journal of psychology*, 10(2), 458-467).

Russian version:

1. В целом я доволен собой
2. Временами мне кажется, что я не совсем хорош
3. Думаю, у меня есть ряд достоинств
4. Многие вещи я способен делать не хуже большинства других людей
5. Мне кажется, что мне нечем гордиться
6. Иногда я чувствую себя бесполезным
7. Я считаю себя достойным и равным другим людям человеком
8. Мне бы хотелось больше уважать себя,
9. По большому счету я считаю себя неудачником
10. Я хорошо отношусь к себе,

Response options:

1. Совершенно согласен
2. Согласен
3. Не согласен
4. Совершенно не согласен

Zolotareva, A. A. (2020). Validity and reliability of the Russian version of the Rosenberg self-esteem scale. *Herald of Omsk University. Series: Psychology*, *2*(2), 52–57. https://doi.org/10.24147/2410-6364.2020.2.52-57

English version:

1. I feel that I am a person of worth, at least on an equal plane with others
2. I feel that I have a number of good qualities

3. All in all, I am inclined to feel that I am a failure
4. I am able to do things as well as most of other people
5. I feel I do not have much to be proud of
6. I take a positive attitude towards myself
7. On the whole, I am satisfied with myself
8. I wish I could have more respect for myself
9. I certainly feel useless at times
10. At times I think I am not good at all

Response options:

1. Strongly agree
2. Agree
3. Disagree
4. Strongly disagree

Table S1. Internal consistency and factorability of the study scales by country.

Scale	Country	No. of items	Sample size (n)	Cronbach's α	KMO	Bartlett's χ^2	Bartlett df	Bartlett p-value	First eigenvalue	Second eigenvalue	Variance explained by first factor (%)
Generalized Trust	Mexico	6	422	0.80	0.85	644.9	15	<0.001	3.01	0.72	50.1
Generalized Trust	Russia	6	254	0.77	0.83	369.3	15	<0.001	2.87	0.84	47.8
Network Orientation Scale	Mexico	20	422	0.70	0.86	1492.5	190	<0.001	6.48	2.21	32.4
Network Orientation Scale	Russia	20	254	0.82	0.84	1110.4	190	<0.001	7.24	2.44	36.2
Rosenberg Self-Esteem Scale	Mexico	10	422	0.88	0.90	1888.5	45	<0.001	4.95	1.13	49.5
Rosenberg Self-Esteem Scale	Russia	10	254	0.88	0.90	1151.7	45	<0.001	5.01	1.10	50.1
Interpersonal	Mexico	15	422	0.78	0.82	1512.1	105	<0.001	4.11	1.68	27.4

Sympathy Scale											
Interpersonal Sympathy Scale	Russia	15	254	0.84	0.85	1311.1	105	<0.001	5.11	1.49	34.1

Cronbach's α evaluates the internal consistency of each scale. The Kaiser-Meyer-Olkin (KMO) statistic assesses sampling adequacy for factor analysis, whereas Bartlett's test evaluates whether the item correlation matrix significantly differs from an identity matrix. The first and second eigenvalues and the percentage of variance explained correspond to the first unrotated factor extracted separately within each country. Because these analyses were conducted independently within each country, they are intended to evaluate within-country psychometric properties rather than direct cross-national differences..

Table S2. Cross-national factorial congruence between Mexico and Russia.

Scale	Items	Tucker's φ	Mean absolute loading difference	Interpretation
Generalized Trust	6	0.94	0.09	Very high similarity
Network Orientation Scale	20	0.88	0.11	Moderate similarity
Rosenberg Self-Esteem Scale	10	0.96	0.07	Virtually identical
Interpersonal Sympathy	15	0.87	0.12	Moderate similarity

Tucker's coefficient of congruence evaluates the similarity of factor loading patterns between the Mexican and Russian samples. Values above 0.90 indicate very high similarity of latent structures. Mean absolute loading difference represents the average absolute difference in standardized factor loadings between countries.

Table S3. Means, standard deviations, and cross-national comparisons of scale scores.

**Scale**	**Mexico Mean**	**Mexico SD**	**Russia Mean**	**Russia SD**	**Welch's t**	**p**	**Hedges' g**
Generalized Trust	2.90	0.69	3.16	0.68	−4.85	<0.001	−0.38
Network Orientation	2.61	0.39	2.58	0.49	0.96	0.335	0.08
Self-Esteem	2.37	0.25	2.72	0.24	−18.16	<0.001	−1.42
Interpersonal Sympathy (Linking People Scale, LPS)	2.64	0.55	2.61	0.66	0.64	0.521	0.05

Means represent average item scores for each scale. Higher values indicate higher levels of the corresponding construct. Between-country comparisons were performed using Welch's unequal-variance t-test. Hedges' g represents the standardized mean difference between the Mexican and Russian samples.

Table S4. Correlation matrix between psychological scales in Mexico.

Scale	Trust	Network Orientation	Self-Esteem	Sympathy
Generalized Trust	1.00	−0.35	−0.06	−0.22
Network Orientation	−0.35	1.00	0.04	0.41
Self-Esteem	−0.06	0.04	1.00	−0.03
Interpersonal Sympathy (Linking People Scale, LPS)	−0.22	0.41	−0.03	1.00

Values represent Pearson correlation coefficients estimated within the Mexican sample (n = 422).

Table S5. Correlation matrix between psychological scales in Russia.

Scale	Trust	Network Orientation	Self-Esteem	Sympathy
Generalized Trust	1.00	−0.50	0.16	−0.22
Network Orientation	−0.50	1.00	−0.29	0.37
Self-Esteem	0.16	−0.29	1.00	−0.04
Interpersonal Sympathy (Linking People Scale, LPS)	−0.22	0.37	−0.04	1.00
Values represent Pearson correlation coefficients estimated within the Russian sample (n = 254).				
